# Effect of non‐alcoholic beer containing matured hop bitter acids on mood states in healthy adults: A single‐arm pilot study

**DOI:** 10.1111/nhs.12898

**Published:** 2021-12-09

**Authors:** Takafumi Fukuda, Shiori Akiyama, Kazuyuki Takahashi, Yasuo Iwadate, Yasuhisa Ano

**Affiliations:** ^1^ Kirin Central Institute, Kirin Holdings Company, Limited Fujisawa Japan; ^2^ Kashiwa‐no‐ha Open Innovation Lab Kashiwa Japan; ^3^ Department of Neurological Surgery Chiba University Graduate School of Medicine Chiba Japan

**Keywords:** hops, mental health, mood, non‐alcoholic beer, nutritional supplements, presenteeism, sleep, stress

## Abstract

This study aimed to investigate the effect of non‐alcoholic beer containing matured hop bitter acids on mood states among healthy adults older than 20 years. This study was an open‐label longitudinal intervention design in which each participant served as their control. For 3 weeks, we evaluated the effect of non‐alcoholic beer containing 35 mg of matured hop bitter acids on mood, sleep quality, and work performance. The data of 97 participants (age range: 23–72 years, median age: 42) were analyzed. After the intervention, we found that matured hop bitter acids significantly improved total mood state, including anxiety, depression, fatigue, and vigor, compared with the baseline. Furthermore, sleep quality and absolute presenteeism were significantly improved after the intervention compared with the baseline. The present exploratory study suggested that 3‐week supplementation with matured hop bitter acids improved mood and peripheral symptoms in persons of a wide range of ages. Although further investigation is needed, the findings suggested that non‐alcoholic beer in daily life might become a choice for maintaining mood states.

**Key Points**

- Supplementation with non‐alcoholic beer containing matured hop bitter acids, bitter components in beer for 3 weeks improved mood states in healthy individuals.
- Sleep quality and work performance were also improved by supplementation with non‐alcoholic beer containing matured hop bitter acids. Although work performance was weak, it was significantly correlated with some mood states.
- Non‐alcoholic beer with matured hop bitter acids can be consumed easily, and might be an effective solution to maintaining mental health.

## 1 INTRODUCTION

Mental health is a topic of interest, given the ubiquitous presence of stress in society. A 2017 survey indicated that over 970 million people worldwide experience mental disorders, including depressive and anxiety disorders (James et al., 2018). According to the report, mental health problems are not confined to a particular age but extend to a wide range of ages. In addition, mental health problems are increasingly becoming a major social issue due to the difficult situations caused by the COVID‐19 pandemic (Yao et al., 2020). Chronic mental health problems lead to mood disorders, including anxiety and depression; therefore, it is important to address mental health issues quickly before the problems become serious. Engaging in self‐care practices, such as healthy eating habits, is recommended to prevent mental health problems during the pandemic (Damiano et al., 2021).

Nutritional intervention in daily life and nutrition‐based approaches for the management of mental health have garnered much attention. Hops (*Humulus lupulus* L.), which impart bitterness and aroma to beer and non‐alcoholic beer, have been reported to have various biological activities, including an improved mood state. Our group previously demonstrated that 12 weeks supplementation of matured hop bitter acids (MHBAs), which are hop‐derived bitter components, improved mood states in middle‐aged and older adults. However, the efficacy of MHBAs in younger individuals and following short‐term supplementation remains unknown.

## 2 BACKGROUND

Research shows that proper dietary habits may prevent mood disorders (Quirk et al., 2013), and particular nutrients improve mood states. For example, supplementation with L‐tryptophan, which is a precursor of serotonin, contributes to the suppression of depressed and anxious moods, and L‐theanine, which is an amino acid present in green tea, also assists in the reduction of stress and anxiety (Okereke et al., 2020; Williams et al., 2020). However, evidence of relationships between mood states and food remains inadequate.

Non‐alcoholic beer is often consumed for its health benefits (Sánchez‐Muniz et al., 2019; Trius‐Soler et al., 2020). A previous study showed that drinking non‐alcoholic beer with supper improved the sleep quality of healthy individuals (Franco et al., 2012; Franco et al., 2014). However, there has been insufficient scientific validation of the efficacy of non‐alcoholic beer and its ingredients regarding its effect on mood states.

The herb hops impart bitterness and aroma to beer and non‐alcoholic beer. MHBAs, which are hop‐derived bitter components, are produced by the oxidation of α‐ and β‐acids (Taniguchi et al., 2013). We previously demonstrated that MHBAs resulted in increasing norepinephrine in the brain and an improvement of depression states. The efficacy of MHBAs was attenuated in vagotomized mice, whose vagus nerves were cut under the diaphragm. Therefore, MHBAs activated the vagus nerve, which improved depression states via a brain‐gut interaction in an animal study (Fukuda, Ohya, et al., 2019). In general, activating the afferent vagus nerve is effective in improving mood states (Breit et al., 2018); however, few food ingredients are known to improve mood states using this strategy.

Furthermore, dopaminergic activity in the hippocampus and middle prefrontal cortex is activated by MHBAs supplementation, which suggests that MHBAs may help alleviate the effects of social stress (Ano et al., 2020). Moreover, clinical studies demonstrated that supplementation with MHBAs (35 mg/day) for 12 weeks in middle‐aged and older adults improved total mood state, especially anxiety, as evaluated by the Profile of Mood States 2nd edition (POMS2); reduced feelings of fatigue, as evaluated by a visual analog scale; and reduced stress response (Fukuda, Obara, et al., 2020; Fukuda, Ohnuma, et al., 2020). Considering the mechanism, that MHBAs controlled neurotransmitters via vagus nerves and resulted in an improvement of mood states, the effect of MHBA should not be limited to middle‐aged and older adults and is expected to be effective in a shorter intervention (Elger et al., 2000; George et al., 2005, 2008; Rush et al., 2005; Schlaepfer et al., 2008). Preclinical studies have confirmed efficacy in young mice for 1 week of intake (Fukuda, Ohya, et al., 2019). However, the efficacy of MHBAs in younger individuals and following short‐term supplementation remains unknown. Therefore, this pilot study aimed to determine whether non‐alcoholic beer containing MHBAs supplementation for 1–3 weeks can improve mood states in healthy adults of all ages in an open‐label longitudinal intervention design where each participant was their control. In addition, work performance, sleep quality, and subjective cognitive function associated with mood states were assessed.

## 3 METHODS

### 3.1 Study design

**FIGURE 1:**
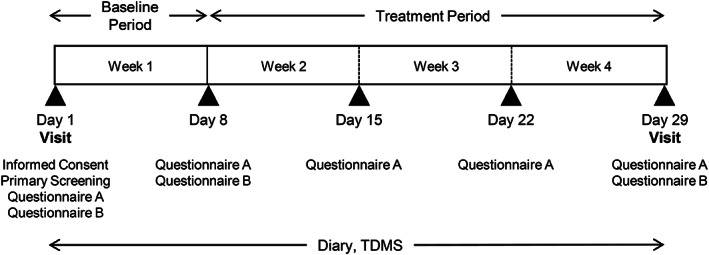
Study flow. After the baseline period, participants had 350 ml of non‐alcoholic beer containing 35 mg MHBAs during the treatment period. Participants completed paper diaries and the Two‐Dimensional Mood Scale (TDMS) every day. Assessments were performed on Days 1, 8, 15, 22, and 29. Questionnaire A contains the brief Profile of Mode States 2nd edition, the Apathy Scale, and the Effortful Control Scale for adults. Questionnaire B contains the short form of the World Health Organization Health and Work Performance Questionnaire and the Pittsburgh Sleep Quality Index, Japanese version

This open‐label, single‐arm pilot study was conducted in Kashiwa City, Chiba Prefecture, Japan. The experimental flow is illustrated in Figure 1. Primary screening to confirm eligibility in terms of inclusion/exclusion criteria was performed on Day 1. This study was composed of a baseline period of 1 week and a treatment period of 3 weeks. Our preclinical study showed that MHBA improved depressive‐like behavior in mice after a 7‐day administration (Fukuda, Ohya, et al., 2019). In addition, other in vivo studies of ours indicated that the possible mechanism of MHBA is to increase neurotransmitters, such as norepinephrine or acetylcholine (Ayabe et al., 2018; Fukuda, Ayabe, et al., 2019). Therefore, we hypothesized the short‐term effects of MHBA and set the length of the treatment period at 3 weeks, including each 7‐day evaluation of the effects of the intervention. During the baseline period, participants did not consume any test food used for this study. After the baseline period, participants drank 350 mL of non‐alcoholic beer containing MHBAs (35 mg; Kirin Brewery Company, Limited, Tokyo) per day during the treatment period. The company gave permission to use the non‐alcoholic beer. Throughout the study, participants completed the Two‐Dimensional Mood Scale (TDMS) every day and two questionnaires: questionnaire A, which consisted of the POMS2 (brief form), the Apathy Scale, and the Effortful Control Scale for adults (ECS) and was completed on Days 1, 8, 15, 22, and 29; and questionnaire B, which consisted of the World Health Organization Health and Work Performance Questionnaire (short form; WHO‐HPQ) and the Pittsburgh Sleep Quality Index, Japanese version (PSQI‐J) and was completed on Days 1, 8, and 29.

### 3.2 Participants

We recruited participants using advertisements in which inclusion and exclusion criteria were listed. The flyers were displayed in Kashiwa‐no‐ha Open Innovation Lab (KOIL; Mitsui Fudosan, Tokyo, Japan) and sent to email newsletter subscribers of KOIL from August to September 2020. We recruited 102 Japanese‐speaking healthy adults, aged 20 years or older, with self‐awareness of mental fatigue. The exclusion criteria were as follows: (i) difficulty obtaining informed consent; (ii) allergy to the non‐alcoholic beer; (iii) pregnancy or trying to get pregnant; (iv) current treatment for any mental or neurological disorder; and (v) the individual or their family working for Kirin Holdings Company or related companies. Family history or past medical history of mental or neurological disorders of the participant were not asked. The inclusion and exclusion criteria were assessed using a questionnaire during the primary screening step. Data were collected using online questionnaires (Questionnaires A and B) or paper diaries (TDMS, compliance, adverse events, alcohol consumption, medicine intake, and unusual incidents if these happen). Participants received 5000 yen as an incentive, regardless of whether they completed the study.

### 3.3 Intervention

The non‐alcoholic beer contained 35 mg of MHBAs and was prepared by Kirin Holdings Company (Tokyo, Japan). During the treatment period (Days 8–28), participants ingested 350 mL of non‐alcoholic beer daily. They were also asked to complete each questionnaire before drinking the non‐alcoholic beer to avoid acute effects. Compliance was assessed using the participants' diaries.

### 3.4 Assessments

#### 3.4.1 Background data

Baseline assessments were made of age, gender, Alcohol Use Disorders Identification Test, and employment status. Additionally, assessments were also made for the frequency of drinking beer or non‐alcoholic beer (never drank/less than once a month/2–4 times a month/2–3 times a week/more than four times a week), and preference for beer or non‐alcoholic beer, respectively. These were measured using a 5‐point Likert scale (0 = dislike non‐alcoholic beer or beer through 4 = like non‐alcoholic beer or beer).

#### 3.4.2 Primary outcomes

The POMS2 and TDMS were used to assess participants' mood states (Konuma et al., 2015; Sakairi et al., 2013). The POMS2 can measure the following seven mood aspects experienced in the preceding week: Anger–Hostility (AH), Confusion–Bewilderment (CB), Depression–Dejection (DD), Fatigue–Inertia (FI), Tension–Anxiety (TA), Vigor–Activity (VA), and Friendliness (F). A total mood disturbance score (TMD) was also calculated, which is a summary measure of distress with higher scores indicating increased mood disturbance, as follows: TMD = (AH + CB + DD + FI + TA) ‐ VA.

The TDMS consists of eight mood‐expressing words related to pleasure and arousal states: energetic, lively, lethargic, listless, relaxed, calm, irritated, and nervous. Participants reported their present psychological mood state every day by rating each item using a 6‐point Likert scale. The average weekly scores of vitalities, stability, pleasure (= vitality + stability), and arousal (= vitality ‐ stability) levels were determined.

#### 3.4.3 Secondary outcomes

The ECS consists of 35 items rated on a 4‐point Likert scale (Yamagata et al., 2005). The scores of the following three subscales were determined according to the original report: Inhibitory Control (IC), Attention Control (AC), and Activation Control (AcC). The global score (GS) was calculated as the total score of all items. Higher scores indicated greater effortful control.

#### 3.4.4 Exploratory outcomes

The Apathy Scale consists of 14 items, rated on a 4‐point Likert scale (Okada et al., 1998). The total score ranges from 0 to 42, with a higher score indicating greater apathy. A score of 16 or above has been used to detect an apathetic profile for Japanese people.

The PSQI‐J consists of 19 items rated on a 4‐point Likert scale. The scale generates the following seven component scores: sleep quality, sleep latency, sleep duration, habitual sleep efficiency, sleep disturbance, use of sleeping medication, and daytime dysfunction. The GS, ranging from 0 to 21, is calculated as the total score of the seven component scores (Doi et al., 2000). A higher score indicates poorer sleep quality. Although the questionnaire originally evaluated subjective sleep quality in the past 4 weeks, we asked participants to review their past 3 weeks because of the study design.

The WHO‐HPQ short form consists of 11 questions and statements that describe work experiences (Kessler et al., 2003). This questionnaire is a self‐report instrument designed to estimate the workplace costs of health problems in terms of reduced job performance, sickness absence, and work‐related accidents/injuries. The tool has been shown to have good validity and internal consistency (Kessler et al., 2003). Although the questionnaire originally evaluated subjective working performance in the past 4 weeks, we asked participants to review their past 3 weeks because of the study design. The WHO‐HPQ can measure two components of work productivity loss: absenteeism, which refers to missing working days due to illness or disability, and presenteeism, which refers to the decreased job performance of employees who are present but not functioning at full capacity due to illness or other medical conditions. The two components consist of relative and absolute evaluations. Briefly, absolute absenteeism is defined as total hours lost from work in a specific time frame. Alternatively, relative absenteeism is hours lost from work relative to the expected work hours. Further, absolute presenteeism is defined as subjective work performance (i.e., the quality of work) rated on a 0–10 scale. In contrast, relative presenteeism is self‐rated work performance relative to that of most co‐workers. The scores of the four components were calculated according to the scoring manual.

### 3.5 Statistical analysis

Data of participants with high compliance (>70%) and without missing values were analyzed. Data are expressed as mean ± SD. Statistical comparisons were performed using Bell Curve for Excel (Social Survey Research Information, Tokyo, Japan) and Microsoft Excel 2016 (Microsoft, Redmond, WA, USA). Data collected on day 8 were used as a baseline. Since the sample size was large enough in this study, we assumed the data normality by central limit theorem (Barron, 1986; Lantz, 2013; Oberfeld & Franke, 2013; Sullivan et al., 2016). The homogeneity of variance and the sphericity of the data were assessed using Bartlett's test and Mauchly's test, respectively. All results were compared using one‐way repeated measures analysis of variance (ANOVA) followed by Bonferroni‐corrected post‐hoc multiple comparisons of absolute values. In addition, a paired *t*‐test was conducted to compare the results between the baseline and Day 29 for the PSQI‐J and the WHO‐HPQ. Spearman's rank correlation coefficients were used for correlation analysis. A McNemar test was conducted to compare the categorical data, and the statistical significance was set at *P* < 0.05. The effect size *d*_*D*_, Equation (1), was calculated according to the following (Glass et al., 1981):

(1) $$d_{D}=\frac{M_{D}}{s_{D}}\;$$

(2) $$x_{D}=x_{1}-x_{2}\;$$

(3) $$M_{D}=\frac{x_{D}}{N}\;$$

(4) $$s_{D}=\sqrt{s_{1}^{2}+s_{2}^{2}-2s_{12}}\;$$

where *x*_1_ and *x*_2_ are the scores of the measurement points to be compared for the same participant in Equation (2); *N* is the number of participants in Equation (3); *S*_1_^2^ and *S*_2_^2^ are the variances of the scores of the measurement points to be compared, and *S*_12_ is the covariance of both measurement points in Equation (4).

### 3.6 Ethics, registrations, and participant consent

The study was conducted following the Declaration of Helsinki and Ethical Guidelines for Medical and Health Research Involving Human Subjects and was approved by the ethics committee of Kirin Holdings Company. Written informed consent was obtained from all participants. The study was registered in July 2020 in the database of the University Hospital Medical Information Network prior to participant enrollment (Registration No. UMIN00004111).

## 4 RESULTS

### 4.1 Participants

Participants were screened, and interventions were conducted between August and September 2020. A total of 102 participants who met the eligibility criteria were recruited, and 100 participants completed the entire study. Two participants were dropped because we were unable to follow up. In addition, three participants were excluded from the analysis because of low compliance (<70%); thus, the data of 97 participants in total were analyzed, and the characteristics of these participants are presented in Table 1. The age range was 23–72 years, with an average age of 44.0, and 89 of 97 were employed. There was no significant difference in the number of working days per week during the study period (Table S1 in the Supporting Information). There were no significant differences in supplementation compliance in week‐to‐week comparisons (Table S2). No serious adverse effects were observed.

**TABLE 1:** Characteristics of the participants

Characteristics	
Age, mean ± SD (range)	44.0 ± 11.9 (23–72)
Female/male	50/47
Employed/unemployed	89/8
Number of working days/week, mean ± SD	4.7 ± 0.9
Working hours/day, mean ± SD	7.6 ± 2.2
AUDIT score, mean ± SD	5.0 ± 4.1
Frequency of beer drinking (A/B/C/D)^a^	0/32/24/17/19
Frequency of non‐alcoholic beer drinking (A/B/C/D)^a^	0/58/12/8/4
Preference for beer (E/F/G/H/I)^b^	47/24/19/5/5
Preference for non‐alcoholic beer (E/F/G/H/I)^b^	16/19/60/3/1

Abbreviation: AUDIT, Alcohol Use Disorders Identification Test.

^a^ A/B/C/D, never drink/less than once a month/2–4 times a month/2–3 times a week/more than four times a week.

^b^ E/F/G/H/I, 4(like)/3/2 (neither like nor dislike)/1/0 (dislike).

### 4.2 Primary outcomes

#### 4.2.1 Mood states assessed by the POMS2 and TDMS

Before ANOVA analysis, we assumed the data normality due to the large sample size and applied Greenhouse–Geisser to compensate for violations of sphericity. TMD scores on Days 15, 22, and 29 were significantly lower than those at baseline (Table 2). Furthermore, all subcategories of the POMS2 improved significantly compared with the baseline at any given time point. In particular, the CB, FI, VA, and F scores showed significant improvement at all time points after the first week of intervention (Table 2). There were no significant differences between Days 1 and 8, except for DD, TA, and TMD. Nevertheless, the *p*‐values before intervention were higher than those after intervention for TMD. In the TDMS, the vitality, stability, and pleasure scores were significantly improved at Weeks 2, 3, and 4 compared with the baseline (Table 3). On the other hand, no significant differences were observed in arousal scores.

**TABLE 2:** Profile of Mood States 2nd edition scores

		Day 1	Day 8	Day 15	Day 22	Day 29	One‐way repeated‐measures ANOVA				
							Sum of squares	Adjusted df	*F*	*P*	$\eta_{p}^{2}$
AH	Mean ± SD	49.4 ± 8.9	48.0 ± 8.1	47.1 ± 7.0	46.6 ± 7.1	45.4 ± 6.4	851.4	3.4	8.59	<0.001	0.085
	*P* (vs Day 8)	0.545	‐	1.000	0.602	0.004					
CB	Mean ± SD	53.5 ± 8.8	51.8 ± 8.7	48.8 ± 7.6	49.2 ± 8.2	47.5 ± 7.7	2169.8	2.9	19.5	<0.001	0.175
	*P* (vs Day 8)	0.285	‐	0.001	0.009	<0.001					
DD	Mean ± SD	51.6 ± 8.9	49.5 ± 7.8	48.0 ± 6.9	47.9 ± 7.0	46.8 ± 6.6	1238.5	2.8	15.2	<0.001	0.142
	*P* (vs Day 8)	0.017	‐	0.301	0.182	<0.001					
FI	Mean ± SD	51.9 ± 9.3	51.0 ± 9.9	48.0 ± 8.5	48.2 ± 9.3	44.8 ± 8.1	2907.9	3.3	25.3	<0.001	0.216
	*P* (vs Day 8)	1.000	‐	0.002	0.005	<0.001					
TA	Mean ± SD	51.1 ± 8.6	48.8 ± 8.7	47.9 ± 7.2	46.9 ± 7.6	45.7 ± 6.8	1575.0	3.2	14.8	<0.001	0.139
	*P* (vs Day 8)	0.027	‐	1.000	0.108	<0.001					
VA	Mean ± SD	48.6 ± 8.6	49.6 ± 8.2	52.9 ± 8.1	54.7 ± 9.0	54.8 ± 8.7	3114.0	3.5	31.1	<0.001	0.253
	*P* (vs Day 8)	1.000	‐	<0.001	<0.001	<0.001					
F	Mean ± SD	48.4 ± 7.3	48.1 ± 6.8	50.4 ± 7.2	52.0 ± 8.2	51.9 ± 8.2	1275.8	3.4	12.7	<0.001	0.122
	*P* (vs Day 8)	1.000	‐	0.020	<0.001	<0.001					
TMD	Mean ± SD	51.8 ± 8.3	49.8 ± 8.1	47.1 ± 6.7	46.6 ± 7.9	44.6 ± 7.4	2952.7	2.7	37.3	<0.001	0.289
	*P* (vs Day 8)	0.026	‐	<0.001	<0.001	<0.001					

*Note*: *P* (vs Day 8) calculated using Bonferroni correction for multiple comparisons.

Abbreviations: AH, anger–hostility; ANOVA, analysis of variance; CB, confusion–bewilderment; DD, depression–dejection; F, Friendliness; FI, fatigue–inertia; VA, vigor–activity; TA, tension–anxiety; TMD, total mood disturbance.

**TABLE 3:** Two‐dimensional mood scale scores

		Week 1	Week 2	Week 3	Week 4	One‐way repeated‐measures analysis of variance				
						Sum of squares	Adjusted df	*F*	*P*	$\eta_{p}^{2}$
Vitality	Mean ± SD	0.0 ± 2.7	0.7 ± 2.4	1.5 ± 2.5	1.9 ± 2.7	216.8	2.2	31.6	<0.001	0.248
	*P* (vs Week 1)	‐	0.004	<0.001	<0.001					
Stability	Mean ± SD	1.7 ± 2.3	2.4 ± 2.3	3.0 ± 2.5	3.4 ± 2.5	158.4	2.3	25.2	<0.001	0.290
	*P* (vs Week 1)	‐	0.004	<0.001	<0.001					
Pleasure	Mean ± SD	1.7 ± 4.3	3.1 ± 3.7	4.5 ± 4.1	5.3 ± 4.4	738.1	2.3	40.4	<0.001	0.248
	*P* (vs Week 1)	‐	<0.001	<0.001	<0.001					
Arousal	Mean ± SD	−1.7 ± 2.5	−1.7 ± 2.9	−1.5 ± 2.8	−1.5 ± 2.8	5.8	2.3	0.7	0.509	0.290
	*P* (vs Week 1)	‐	1.000	1.000	1.000					

*Note*: *P* (vs Week 1) calculated using Bonferroni correction for multiple comparisons.

### 4.3 Secondary outcomes

#### 4.3.1 Subjective cognitive function assessed by the effortful control scale for adults

To assess subjective cognitive function, the ECS was administered. GS and AC scores on Days 15, 22, and 29 were significantly higher than those at baseline (Table S3). Additionally, the IC and AcC scores on Days 22 and 29 were significantly improved compared to baseline. Significant differences between Days 1 and 8 were not observed in any of the categories.

### 4.4 Exploratory outcomes

#### 4.4.1 Apathy state

**FIGURE 2:**
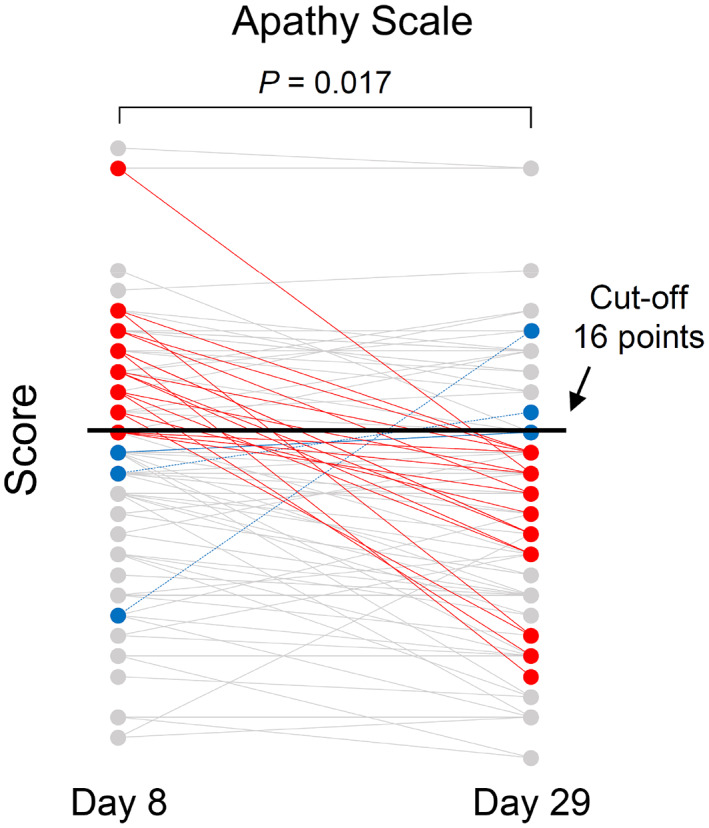
Individual Apathy Scale scores on Days 8 and 29. The cut‐off value of the Apathy Scale is 16 points. The red lines represent the 19 participants whose scores were 16 or higher at baseline and less than 16 on Day 29 (McNemar test: χ^2^ = 5.65, *P* = 0.017). The blue dotted lines represent the four participants whose scores were less than 16 at baseline and above 16 on Day 29

The Apathy Scale score significantly improved on Days 22 and 29 compared to baseline (Table S4). In clinical practice, a score of 16 or higher on the Apathy Scale is defined as a low motivation state. At baseline, the number of participants with a score of 16 or higher was 44, but after the intervention, the number decreased to 28 on Day 29 (χ^2^ = 5.65, *P* = 0.017; Figure 2).

#### 4.4.2 Sleep quality

Sleep quality as assessed by PSQI‐J was significantly improved at Day 29 compared to baseline (Table S5).

#### 4.4.3 Work performance

Work performance was assessed using the WHO‐HPQ. Absolute presenteeism was significantly higher after the intervention than at baseline (Table S6). We did not observe any differences between post‐intervention and baseline for the other scores, namely for absolute absenteeism, relative absenteeism, and relative presenteeism. A weak but significant correlation was observed between absolute presenteeism and CB, DD, and sleep quality (Spearman's rank correlation coefficient: *r*_*s*_ = −0.31, *P* = 0.004; *r*_*s*_ = −0.27, *P* = 0.014; *r*_*s*_ = −0.32, *P* = 0.004, respectively; Figure S1).

### 4.5 Correlations between drinking habits, preference for non‐alcoholic beer and changes in drinking frequency and POMS2 scores

Correlation analysis was performed to investigate the relationship between drinking habits, preference for non‐alcoholic beer, and changes in drinking frequency from Week 1 to Week 2 or 4 calculated from paper diaries, and the efficacy of non‐alcoholic beer containing MHBAs in terms of changes in the POMS2 (Table S7). Spearman's rank correlation coefficients for all comparisons were less than 0.30.

### 4.6 Correlations between age and the efficacy of MHBAs

Correlation analysis was performed to investigate the relationship between age and the efficacy of MHBAs (Table S8). Spearman's rank correlation coefficients for all comparisons were less than 0.30.

### 4.7 Subgroup analysis by age

We conducted a subgroup analysis of primary outcomes classified by participants' median age (42 years) according to the predetermined study protocol. As shown in Table S9, in the 42 years or older group, TMD scores on Day 29, VA and F on Days 15 and 29, and CB and FI on Day 29 of the POMS2 were significantly improved compared with baseline. In the younger group, TMD and CB scores on Days 15 and 29, and DD, TA, FI, VA, and F on Day 29 were significantly improved compared with baseline. To compare these results with the overall analysis, the effect sizes on Days 15 and 29 were calculated (Table S9). The effect size for the TMD did not differ between groups (older group: *d*_*D*_ [Day 15] = −0.53, *d*_*D*_ [Day 29] = −0.65; younger group: *d*_*D*_ [Day 15] = −0.37, *d*_*D*_ [Day 29] = −0.71).

## 5 DISCUSSION

In this open‐label single‐arm pilot study, we evaluated the effect of MHBAs supplementation (35 mg/day) for 1–3 weeks on mood state in healthy adults. Furthermore, effects were assessed on subjective cognitive function, sleep quality, and work performance. Of primary outcomes, all categories of the POMS2 and TDMS, except for arousal, were significantly improved after the intervention compared with baseline. Arousal was defined by the difference between vitality and stability and was described as a reference value in the original paper (Sakairi et al., 2013). Since MHBAs increased both vitality and stability, it is probable that there was no change in arousal.

Significant differences were observed in CB, FI, VA, and F of the POMS2 and vitality, stability, and pleasure of the TDMS from 1 week after the intervention, suggesting that MHBAs are particularly effective for these domains of mood state. A previous preclinical study indicated that MHBAs improved mood states by activating the vagus nerve and increasing norepinephrine in the hippocampus (Fukuda, Ayabe, et al., 2019) and activating dopaminergic systems in the hippocampus and middle prefrontal cortex (Ano et al., 2020). Since decreased norepinephrine and dopamine causes fatigue, low energy, and lack of motivation (Stahl, 2002), MHBAs probably improve fatigue and vigor in short‐term supplementation by replenishing these neurotransmitters. In addition, even though significant differences between Days 1 and 8 were observed only in DD, TA, and TMD, we know that most of the items tend to show positive changes from Day 1 to 8 (i.e., placebo effects). However, for CB, FI, VA, and F, there was no significant difference between Days 1 and 8.

In contrast, there were significant differences between Days 8 and 15. Furthermore, a significant difference in TMD was observed in the first week, but the *P*‐values after the intervention were smaller. These findings indicate that the effect of MHBAs supplementation was greater than the placebo effect. The POMS2 results showed that the effects of MHBAs appear later for anxiety and depression than for fatigue and vitality. These results suggested that the mechanism of action for anxiety and depression might include an increase in neurotransmitters and, for example, suppression of inflammation, which need a relatively long time. In future studies, we will investigate the effects of longer‐term interventions.

Although the POMS2 is occasionally used for pre‐post comparison design trials as in this study (Biersack et al., 2016; Lane et al., 2007; Tanaka et al., 2018), in the future, placebo‐controlled trials are required to evaluate the efficacy of MHBAs without the influence of the placebo effect.

A previous randomized controlled trial of healthy middle‐aged and older adults (45–65 years old) demonstrated that total mood states and fatigue after a 6‐week supplementation of MHBAs and anxiety after 12‐week supplementation were improved in an intent‐to‐treat analysis (Fukuda, Obara, et al., 2020). On the other hand, this study demonstrated the effect of a shorter intake period. The acute efficacy of vagus nerve stimulation on fatigue and depression has been reported (Ferstl et al., 2021); therefore, the results of this study are explicable. Furthermore, because of correlation analysis, there was no correlation between age and the efficacy of MHBAs (Table S8). The subgroup analysis classified by median age also did not affect the POMS2 score (Table S9). These results indicated that the efficacy of MHBAs was not only for older adults but also for relatively younger adults. Given the mechanism that increases norepinephrine in the brain, the effects are likely to occur regardless of age.

It was clarified for the first time that sleep quality was significantly improved by MHBAs treatment compared with baseline. The underlying mechanism is unclear, but a previous study indicated that vagus nerve stimulation improved sleep quality in insomnia (Jiao et al., 2020). Previous studies showed that a non‐alcoholic beer intervention with supper for healthy participants improved sleep quality (Franco et al., 2012, 2014). Although reports have considered that hop‐derived components, such as 2‐methyl‐3‐buten‐2‐ol in non‐alcoholic beer, might increase the activity of γ‐aminobutyric acid resulting in sedative effects, generally available non‐alcoholic beer made with hops contains MHBAs. Therefore, the effectiveness in terms of improving sleep quality might be fostered by MHBAs. Further studies are needed to identify the active ingredients associated with improved sleep quality.

MHBAs supplementation also improved apathy, which may be caused by the functional decline of the frontal cortex, including the dorsolateral prefrontal cortex and basal nuclei (Franceschi et al., 2005; Lopez et al., 2001). As mentioned above, MHBAs increase norepinephrine via the locus coeruleus by activating the vagus nerve. Since the neurons of the locus coeruleus project broadly throughout the brain, including the prefrontal cortex, activation of this pathway probably fostered the improvement in apathy. In clinical practice, the Apathy Scale is used to screen for apathy, and the cut‐off value is set to a score of 16 (Okada et al., 1998). As shown in Figure 2, the number of participants whose Apathy Scale score was 16 or higher decreased after the intervention. This result indicated that MHBAs are effective for patients with mild diseases and represent a potential non‐pharmacological treatment for apathy.

Mood disorders impair productivity and induce social losses (Dewa & Hoch, 2015). To investigate whether the improvement in mood states by MHBAs treatment affected work performance, the WHO‐HPQ was conducted. The results showed that the intervention improved absolute presenteeism, and weak but significant correlations were observed between absolute presenteeism and CB, DD, and sleep quality (Figure S1). This finding is consistent with a previous report that showed an association between depression, sleep quality, and productivity (Takami et al., 2018) and suggests that MHBAs improved mood states, resulting in enhanced productivity.

In the present study, non‐alcoholic beer containing MHBAs was used as the intervention. Currently, the health benefits of non‐alcoholic beer consumption are gaining attention (Sánchez‐Muniz et al., 2019; Trius‐Soler et al., 2020). Moreover, it has been suggested that non‐alcoholic beer may also be beneficial for caring for specific diseases such as liver cirrhosis (Macías‐Rodríguez et al., 2020). In this study, no correlations between the changes in the frequency of drinking and changes in POMS2 scores were observed (Table S7). This result suggests that the health benefit of supplementation with non‐alcoholic beer is due to a reduction in alcohol consumption and supplementation of MHBAs derived from hops. In addition, there was a very low correlation (*r*_s_ < 0.30) between drinking habits or preference for non‐alcoholic beer and the efficacy of MHBAs on mood states (Table S7). This indicates that the improvement in mood state was not due to a preference for non‐alcoholic beer. Even though most participants did not have the habit of drinking non‐alcoholic beer, the intake compliance was above 95%, and only three participants exhibited compliance below 70%. Although further study is needed, this result indicates that non‐alcoholic beer rather than the supplement form is suitable for supplying MHBAs in daily life. In this study, we did not instruct the timing for intake of non‐alcoholic beer. Therefore, the most effective intake timing remains unclear. But almost all participants took the test beverage at night; therefore, taking it at night may be effective. To investigate this further is one of our future tasks. Furthermore, some non‐alcoholic beer contains many carbohydrates. Therefore, for health reasons, proper care should be taken when consuming non‐alcoholic beer.

There were some limitations to the present study. First, this study used an open‐label single‐arm design because of the difficulty in preparing a placebo. Because some previous pre‐post comparison design trials assessed mood states using the POMS2 (Biersack et al., 2016; Lane et al., 2007; Tanaka et al., 2018), mood states were probably evaluated appropriately in this study, but the study design was subject to the risk of bias. A placebo effect was observed from Days 1 to 8. In addition, the sample size was relatively small, therefore it may not adequately reflect the target population. For further investigation, a placebo‐controlled study design with a large sample size should be conducted or added to the post‐observation period to confirm the efficacy of MHBAs for short‐term supplementation to persons of a wide range of ages. Second, all outcomes were subjective evaluations using questionnaires. Future studies should include objective biomarker measurements to assess the participants' stress statement, such as salivary cortisol concentrations, chromogranin A, or autonomic response. Third, we collected information on the lives of the participants during the study from a diary. Still, there were limits, and there was a possibility that some factors that we did not understand might affect mood states and other outcomes. Finally, healthy adult men and women participated in this study, but further research is needed to clarify the extent the results can be generalized.

## 6 CONCLUSION

This study suggested the possibility that intake of non‐alcoholic beer rich in MHBAs for 1–3 weeks improves mood states, sleep quality, and work performance. However, this study is a single‐arm study. Therefore, further study is needed to confirm the efficacy of non‐alcoholic beer containing MHBAs, and the effects of overdose are unknown. Therefore, there are some issues to be solved for clinical practice.

## CONFLICT OF INTERESTS

Takafumi Fukuda, Shiori Akiyama, and Yasuhisa Ano are employees of the Kirin Holdings Company, Limited, the study funder. All authors have declared that they have no further conflicts of interest. Kirin Holdings Company, Limited holds patents on the MHBAs and related products.

## AUTHOR CONTRIBUTIONS

Study design: Takafumi Fukuda, Shiori Akiyama, Yasuhisa Ano.

Judgment of adverse events: Yasuo Iwadate.

Data collection: Takafumi Fukuda, Shiori Akiyama, Kazuyuki Takahashi.

Data analysis: Takafumi Fukuda, Shiori Akiyama.

Manuscript writing: Takafumi Fukuda, Shiori Akiyama, Yasuo Iwadate, Yasuhisa Ano.

## Supporting information


**Appendix S1**: Supporting Information

## Data Availability

The data that support the findings of this study are available from the corresponding author upon reasonable request.
