# Supplementary material for: Effect of non‐alcoholic beer containing matured hop bitter acids on mood states in healthy adults: A single‐arm pilot study
Source: Nurs Health Sci. 2021 Dec 9;24(1):7–16. doi: 10.1111/nhs.12898 (PMC9300118; doi:10.1111/nhs.12898)
Supplement: Supplementary file 1 — Appendix S1: Supporting Information [file NHS-24-7-s001.docx]

**Supporting Information**


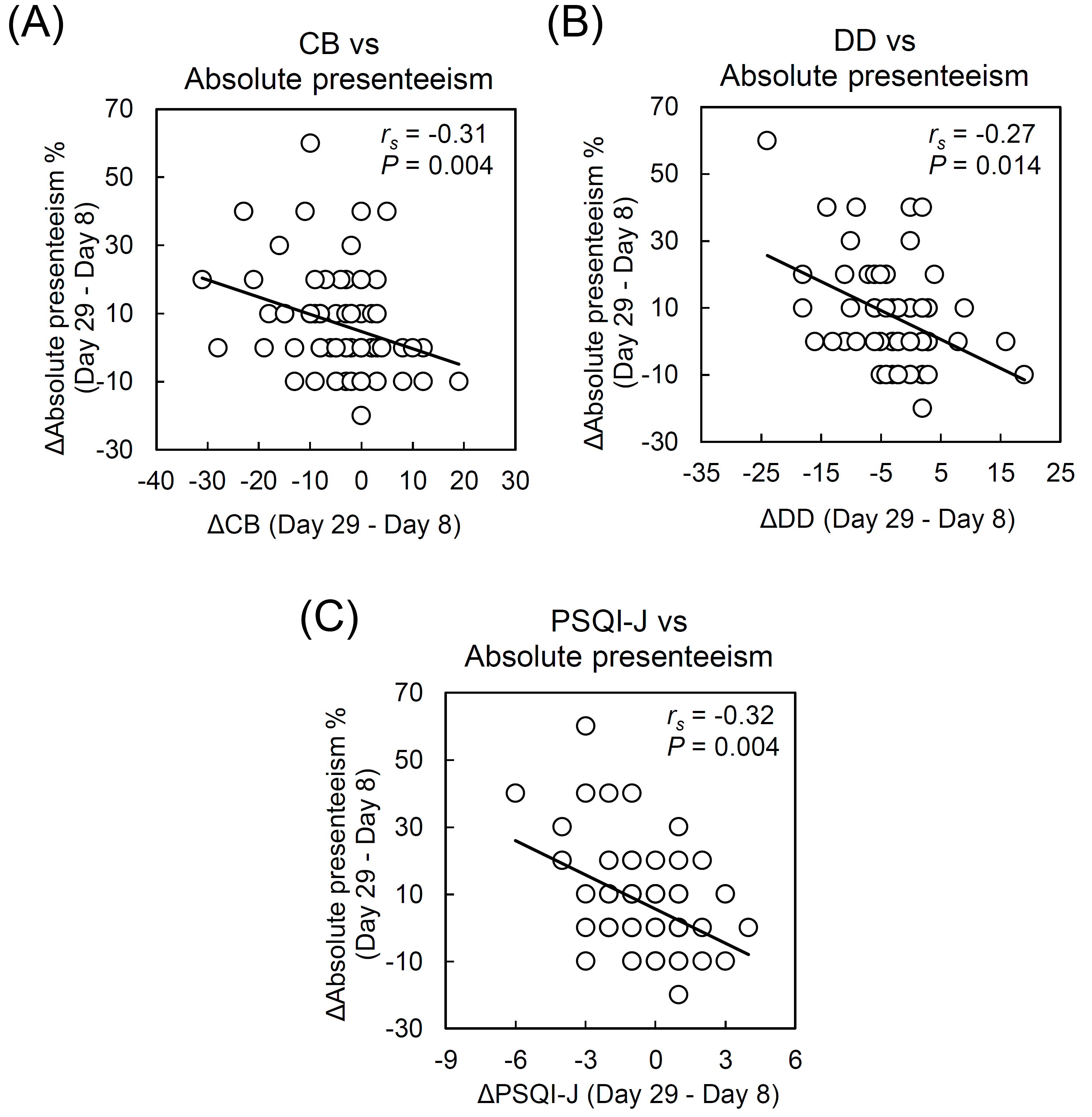


**Supplementary Figure 1** (A) The relationship between change in CB of POMS2 from baseline to day 21 and the change in absolute presenteeism from baseline to day 21 (Spearman's rank correlation coefficient: *r*_s_ = -0.31, *P* = 0.004). (B) The relationship between change in DD of POMS2 from baseline to day 21 and the change in absolute presenteeism from baseline to day 21 (Spearman's rank correlation coefficient: *r*_s_ = -0.27, *P* = 0.014). (C) The relationship between change in PSQI-J from baseline to day 21 and the change in absolute presenteeism from baseline to day 21 (Spearman's rank correlation coefficient: *r*_s_ = -0.32, *P* = 0.004). POMS2, Profile of Mood States 2^nd^ Edition; PSQI-J, Pittsburgh Sleep Quality Index; CB, Confusion–Bewilderment; DD, Depression–Dejection.

**Supplementary Table 1 Working days (days/week)**

|  | Week 1 | | | Week 2 | | | Week 3 | | | Week 4 | | | One-way repeated-measures ANOVA | | | | |
| --- | --- | --- | --- | --- | --- | --- | --- | --- | --- | --- | --- | --- | --- | --- | --- | --- | --- |
|  |  |  |  |  |  |  |  |  |  |  |  |  | Sum of squares | Adjusted df | *F* | *P* | *η_p_^2^* |
| Total subjects | 4.3 | ± | 1.8 | 4.2 | ± | 1.7 | 4.2 | ± | 1.6 | 4.3 | ± | 1.6 | 1.4 | 2.5 | 0.8 | 0.488 | 0.008 |
| Employed subjects | 4.6 | ± | 1.4 | 4.5 | ± | 1.3 | 4.5 | ± | 1.2 | 4.6 | ± | 1.1 | 1.5 | 2.5 | 0.8 | 0.488 | 0.009 |

**Supplementary Table 2 Supplementation compliance**

|  | Week 2 | | | Week 3 | | | Week 4 | | | Total | | | One-way repeated-measures ANOVA | | | | |
| --- | --- | --- | --- | --- | --- | --- | --- | --- | --- | --- | --- | --- | --- | --- | --- | --- | --- |
|  |  |  |  |  |  |  |  |  |  |  |  |  | Sum of squares | Adjusted df | *F* | *P* | *η_p_^2^* |
| Compliance % mean ± SD | 96.0 | ± | 10.0 | 95.1 | ± | 11.0 | 97.0 | ± | 8.2 | 96.0 | ± | 8.2 | 172.8 | 1.8 | 2.1 | 0.134 | 0.020 |

**Supplementary Table 3 Effortful Control Scale for Adults scores**

|  |  | Day 1 | | | Day 8 | | | Day 15 | | | Day 22 | | | Day 29 | | | One-way repeated-measures ANOVA | | | | |
| --- | --- | --- | --- | --- | --- | --- | --- | --- | --- | --- | --- | --- | --- | --- | --- | --- | --- | --- | --- | --- | --- |
|  |  |  |  |  |  |  |  |  |  |  |  |  |  |  |  |  | Sum of squares | Adjusted df | *F* | *P* | *η_p_^2^* |
| IC | Mean ± SD | 31.6 | ± | 4.5 | 32.0 | ± | 4.4 | 32.9 | ± | 4.7 | 33.3 | ± | 4.6 | 33.8 | ± | 4.8 | 299.5 | 3.4 | 13.2 | <0.001 | 0.125 |
|  | *P* (vs. Day 8) | 1.000 | | | - | | | 0.095 | | | 0.004 | | | < 0.001 | | |  |  |  |  |  |
| AcC | Mean ± SD | 33.4 | ± | 5.5 | 33.3 | ± | 5.5 | 34.2 | ± | 5.6 | 34.8 | ± | 5.8 | 35.5 | ± | 6.2 | 331.1 | 3.3 | 17.1 | <0.001 | 0.184 |
|  | *P* (vs. Day 8) | 1.000 | | | - | | | 0.062 | | | < 0.001 | | | < 0.001 | | |  |  |  |  |  |
| AC | Mean ± SD | 29.3 | ± | 5.4 | 30.2 | ± | 5.6 | 31.4 | ± | 5.8 | 32.1 | ± | 6.3 | 32.6 | ± | 6.4 | 697.2 | 3.5 | 21.0 | <0.001 | 0.125 |
|  | *P* (vs. Day 8) | 0.437 | | | - | | | 0.028 | | | < 0.001 | | | < 0.001 | | |  |  |  |  |  |
| GS | Mean ± SD | 94.4 | ± | 12.4 | 95.5 | ± | 12.7 | 98.6 | ± | 13.4 | 100.2 | ± | 13.8 | 101.9 | ± | 14.7 | 3746.1 | 3.1 | 32.1 | <0.001 | 0.184 |
|  | *P* (vs. Day 8) | 1.000 | | | - | | | 0.001 | | | < 0.001 | | | < 0.001 | | |  |  |  |  |  |

IC, Inhibitory Control; AcC, Activation Control; AC, Attention Control; GS, Global Score; *P* (vs. Day 8) calculated with Bonferroni correction for multiple comparisons.

**Supplementary Table 4 Apathy Scale scores**

|  |  | Day 1 | | | Day 8 | | | Day 15 | | | Day 22 | | | Day 29 | | | One-way repeated-measures ANOVA | | | | |
| --- | --- | --- | --- | --- | --- | --- | --- | --- | --- | --- | --- | --- | --- | --- | --- | --- | --- | --- | --- | --- | --- |
|  |  |  |  |  |  |  |  |  |  |  |  |  |  |  |  |  | Sum of squares | Adjusted df | *F* | *P* | *η_p_^2^* |
| Apathy Scale | Mean ± SD | 15.1 | ± | 5.9 | 14.9 | ± | 6.1 | 13.9 | ± | 5.7 | 13.2 | ± | 6.1 | 12.4 | ± | 6.4 | 508.2 | 3.3 | 13.3 | <0.001 | 0.125 |
|  | *P* (vs Day 8) | 1.000 | | | - | | | 0.213 | | | 0.002 | | | < 0.001 | | |  |  |  |  |  |

*P* (vs. Day 8) calculated with Bonferroni correction for multiple comparisons.

**Supplementary Table 5 Pittsburgh Sleep Quality Index, Japanese version scores**

|  | Day 1 | | | Day 8 | | | Day 29 | | | *P* (Day 8 vs Day 29) |
| --- | --- | --- | --- | --- | --- | --- | --- | --- | --- | --- |
| GS | 5.8 | ± | 2.2 | 5.3 | ± | 2.4 | 4.9 | ± | 2.1 | 0.042 |

GS, Global Score; *P* (Day 8 vs. Day 29) calculated by paired *t*-test.

**Supplementary Table 6 World Health Organization Health and Work Performance Questionnaire scores**

|  | Day 1 | | | Day 8 | | | Day 29 | | | *P* (Day 8 vs. Day 29) |
| --- | --- | --- | --- | --- | --- | --- | --- | --- | --- | --- |
| Absolute presenteeism | 63.0 | ± | 16.8 | 64.5 | ± | 18.3 | 71.3 | ± | 15.4 | < 0.001 |
| Relative presenteeism | 1.1 | ± | 0.3 | 1.0 | ± | 0.3 | 1.1 | ± | 0.3 | 0.177 |
| Absolute absenteeism | 4.2 | ± | 38.5 | -4.4 | ± | 46.5 | -12.4 | ± | 40.5 | 0.172 |
| Relative absenteeism | 0.0 | ± | 0.4 | -0.1 | ± | 0.8 | -0.1 | ± | 0.4 | 0.899 |

*P* (Day 8 vs. Day 29) calculated by paired *t*-test.

**Supplementary Table 7 Correlations between drinking habits or preference for non-alcoholic beer and changes in drinking frequency and Profile of Mood States 2^nd^ edition score changes**

|  | Spearman's rank correlation coefficient: *r_s_* | | | | | | | | | | | | | | | | |
| --- | --- | --- | --- | --- | --- | --- | --- | --- | --- | --- | --- | --- | --- | --- | --- | --- | --- |
|  | Changes from Day 8 to Day 15 | | | | | | | |  | Changes from Day 8 to Day 29 | | | | | | | |
|  | AH | CB | DD | FI | TA | VA | F | TMD |  | AH | CB | DD | FI | TA | VA | F | TMD |
| Frequency of non-alcoholic beer drinking usually | 0.10 | 0.04 | 0.25 | 0.25 | 0.13 | 0.01 | -0.12 | 0.18 |  | 0.06 | -0.02 | 0.23 | 0.16 | 0.07 | 0.11 | -0.05 | 0.10 |
| Preference for non-alcoholic beer | -0.07 | -0.06 | 0.04 | 0.16 | 0.00 | -0.05 | -0.06 | 0.02 |  | 0.00 | -0.09 | 0.14 | 0.05 | -0.13 | 0.06 | 0.01 | 0.00 |
| Changes in drinking frequency from week 1 to week 2 or 4 | -0.12 | -0.01 | -0.06 | -0.06 | 0.02 | 0.04 | 0.07 | -0.03 |  | 0.11 | 0.09 | -0.14 | 0.09 | 0.17 | -0.04 | 0.07 | 0.09 |

AH, Anger–Hostility; CB, Confusion–Bewilderment; DD, Depression–Dejection; FI, Fatigue–Inertia; TA, Tension–Anxiety; VA, Vigor–Activity; F, Friendliness; TMD, Total mood disturbance; *r*_s,_ Spearman's rank correlation coefficient.

**Supplementary Table 8 Correlations between the change in score of each questionnaire and age**

| Category | | Period | *r_s_* |  | Category | | Period | *r_s_* |
| --- | --- | --- | --- | --- | --- | --- | --- | --- |
| POMS2 | AH | Day 8-15 | -0.15 |  | TDMS | Vitality | Week 1-2 | 0.02 |
|  |  | Day 8-29 | -0.16 |  |  |  | Week 1-4 | 0.00 |
|  | CB | Day 8-15 | -0.05 |  |  | Stability | Week 1-2 | 0.15 |
|  |  | Day 8-29 | -0.06 |  |  |  | Week 1-4 | 0.23 |
|  | DD | Day 8-15 | -0.04 |  |  | Pleasure | Week 1-2 | 0.07 |
|  |  | Day 8-29 | -0.07 |  |  |  | Week 1-4 | 0.06 |
|  | FI | Day 8-15 | -0.08 |  |  | Arousal | Week 1-2 | -0.11 |
|  |  | Day 8-29 | -0.07 |  |  |  | Week 1-4 | -0.22 |
|  | TA | Day 8-15 | 0.10 |  | Apathy Scale | | Day 8-15 | 0.00 |
|  |  | Day 8-29 | -0.04 |  |  |  | Day 8-29 | -0.13 |
|  | VA | Day 8-15 | 0.10 |  | PSQI-J | | Day 8-29 | 0.03 |
|  |  | Day 8-29 | -0.05 |  | WHO-HPQ | | Day 8-29 | -0.05 |
|  | F | Day 8-15 | 0.11 |  | ECS  (Global score) | | Day 8-15 | 0.01 |
|  |  | Day 8-29 | -0.02 |  |  |  | Day 8-29 | 0.04 |
|  | TMD | Day 8-15 | -0.10 |  |  |  |  |  |
|  |  | Day 8-29 | -0.05 |  |  |  |  |  |

POMS2, Profile of Mood States 2^nd^ edition; AH, Anger–Hostility; CB, Confusion–Bewilderment; DD, Depression–Dejection; FI, Fatigue–Inertia; TA, Tension–Anxiety; VA, Vigor–Activity; F, Friendliness; TMD, Total Mood Disturbance; TDMS, Two-Dimensional Mood Scale; PSQI-J, Pittsburgh Sleep Quality Index, Japanese version; WHO-HPQ, World Health Organization Health, and Work Performance Questionnaire; ECS, Effortful Control Scale for adults; *r_s_*, Spearman's rank correlation coefficient

**Supplementary Table 9 Effect sizes (*d_D_*) for Profile of Mood States subgroup analysis**

| Category | Group |  | Day 1–8 | | |  | Day 8–15 | | |  | Day 8–29 | | |
| --- | --- | --- | --- | --- | --- | --- | --- | --- | --- | --- | --- | --- | --- |
|  |  |  | *d_D_* |  | *P* |  | *d_D_* |  | *P* |  | *d_D_* |  | *P* |
| AH | All |  | -0.18 |  | 0.545 |  | -0.14 |  | 1.000 |  | -0.34 |  | 0.004 |
|  | Age > 42 |  | -0.15 |  | 1.000 |  | -0.32 |  | 0.363 |  | -0.41 |  | 0.014 |
|  | Age < 42 |  | -0.20 |  | 1.000 |  | 0.02 |  | 1.000 |  | -0.26 |  | 0.563 |
| CB | All |  | -0.25 |  | 0.285 |  | -0.42 |  | 0.001 |  | -0.51 |  | <0.001 |
|  | Age > 42 |  | -0.35 |  | 0.264 |  | -0.43 |  | 0.128 |  | -0.47 |  | 0.006 |
|  | Age < 42 |  | -0.13 |  | 1.000 |  | -0.40 |  | 0.034 |  | -0.54 |  | <0.001 |
| DD | All |  | -0.33 |  | 0.017 |  | -0.26 |  | 0.301 |  | -0.38 |  | <0.001 |
|  | Age > 42 |  | -0.44 |  | 0.036 |  | -0.23 |  | 1.000 |  | -0.32 |  | 0.278 |
|  | Age < 42 |  | -0.19 |  | 1.000 |  | -0.28 |  | 0.485 |  | -0.46 |  | 0.004 |
| FI | All |  | -0.12 |  | 1.000 |  | -0.39 |  | 0.002 |  | -0.71 |  | <0.001 |
|  | Age > 42 |  | -0.26 |  | 0.867 |  | -0.47 |  | 0.031 |  | -0.71 |  | <0.001 |
|  | Age < 42 |  | 0.01 |  | 1.000 |  | -0.32 |  | 0.240 |  | -0.70 |  | <0.001 |
| TA | All |  | -0.30 |  | 0.027 |  | -0.13 |  | 1.000 |  | -0.37 |  | <0.001 |
|  | Age > 42 |  | -0.42 |  | 0.034 |  | 0.03 |  | 1.000 |  | -0.34 |  | 0.084 |
|  | Age < 42 |  | -0.17 |  | 1.000 |  | -0.25 |  | 0.472 |  | -0.40 |  | 0.013 |
| VA | All |  | 0.15 |  | 1.000 |  | 0.50 |  | <0.001 |  | 0.67 |  | <0.001 |
|  | Age > 42 |  | 0.14 |  | 1.000 |  | 0.58 |  | <0.001 |  | 0.71 |  | <0.001 |
|  | Age < 42 |  | 0.15 |  | 1.000 |  | 0.40 |  | 0.270 |  | 0.66 |  | <0.001 |
| F | All |  | -0.04 |  | 1.000 |  | 0.35 |  | 0.020 |  | 0.54 |  | <0.001 |
|  | Age > 42 |  | -0.13 |  | 1.000 |  | 0.50 |  | 0.015 |  | 0.52 |  | 0.007 |
|  | Age < 42 |  | 0.07 |  | 1.000 |  | 0.20 |  | 1.000 |  | 0.55 |  | 0.001 |
| TMD | All |  | -0.34 |  | 0.026 |  | -0.45 |  | <0.001 |  | -0.69 |  | <0.001 |
|  | Age > 42 |  | -0.43 |  | 0.0495 |  | -0.53 |  | 0.023 |  | -0.65 |  | <0.001 |
|  | Age < 42 |  | -0.23 |  | 1.000 |  | -0.37 |  | 0.072 |  | -0.71 |  | <0.001 |

AH, Anger–Hostility; CB, Confusion–Bewilderment; DD, Depression–Dejection; FI, Fatigue–Inertia; TA, Tension–Anxiety; VA, Vigor–Activity; F, Friendliness; TMD, Total Mood Disturbance; *d_D_*, Effect size; *P* calculated with Bonferroni correction for multiple comparisons.
